# Enhancing the clinical value of serum neurofilament light chain measurement

**DOI:** 10.1172/jci.insight.161415

**Published:** 2022-08-08

**Authors:** Peter Kosa, Ruturaj Masvekar, Mika Komori, Jonathan Phillips, Vighnesh Ramesh, Mihael Varosanec, Mary Sandford, Bibiana Bielekova

**Affiliations:** Neuroimmunological Diseases Section, Laboratory of Clinical Immunology and Microbiology, National Institute of Allergy and Infectious Diseases, National Institutes of Health (NIH), Bethesda, Maryland, USA.

**Keywords:** Neuroscience, Multiple sclerosis, Neurodegeneration

## Abstract

**BACKGROUND:**

Serum neurofilament light chain (sNFL) is becoming an important biomarker of neuro-axonal injury. Though sNFL correlates with CSF NFL (cNFL), 40% to 60% of variance remains unexplained. We aimed to mathematically adjust sNFL to strengthen its clinical value.

**METHODS:**

We measured NFL in a blinded fashion in 1138 matched CSF and serum samples from 571 patients. Multiple linear regression (MLR) models constructed in the training cohort were validated in an independent cohort.

**RESULTS:**

An MLR model that included age, blood urea nitrogen, alkaline phosphatase, creatinine, and weight improved correlations of cNFL with sNFL (from *R*^2^ = 0.57 to 0.67). Covariate adjustment significantly improved the correlation of sNFL with the number of contrast-enhancing lesions (from *R*^2^ = 0.18 to 0.28; 36% improvement) in the validation cohort of patients with multiple sclerosis (MS). Unexpectedly, only sNFL, but not cNFL, weakly but significantly correlated with cross-sectional MS severity outcomes. Investigating 2 nonoverlapping hypotheses, we showed that patients with proportionally higher sNFL to cNFL had higher clinical and radiological evidence of spinal cord (SC) injury and probably released NFL from peripheral axons into blood, bypassing the CSF.

**CONCLUSION:**

sNFL captures 2 sources of axonal injury, central and peripheral, the latter reflecting SC damage, which primarily drives disability progression in MS.

**TRIAL REGISTRATION:**

ClinicalTrials.gov NCT00794352.

**FUNDING:**

Division of Intramural Research, National Institute of Allergy and Infectious Diseases, NIH (AI001242 and AI001243).

## Introduction

Although many laboratory tests measure injury to peripheral organs, such as liver or kidneys, quantifying central nervous system (CNS) injury with peripheral blood biomarkers is extremely difficult because the concentrations of analytes released during neuronal damage are below detection levels of most of the conventional immunoassays. Two developments in recent time have changed this reality: a) new, advanced immunoassays, such as Single Molecule Array (SIMOA), capable of reliably measuring femtomolar concentrations of proteins, and b) demonstration that neurofilament light chain (NFL), a protein exclusively expressed in neurons, remains elevated for weeks after acute neuro-axonal injury. Indeed, after traumatic brain injury, the serum levels of some neuronal proteins, such as ubiquitin carboxy-terminal hydrolase L1 and neuron-specific enolase, peak at 24 hours and become undetectable 96 hours later, while NFL levels rise gradually, peak in several days, and remain elevated for up to a few weeks (1). This unusual dynamic makes (serum/plasma) NFL an excellent biomarker of acute neuronal injuries, such as those caused by trauma or hypoxia, and for monitoring of chronic neurological diseases, including multiple sclerosis (MS).

Many papers demonstrated associations between CSF and serum NFL levels and formation of acute MS lesions (i.e., contrast-enhancing lesions [CELs] on brain MRI; refs. 2, 3) or an association of therapy-induced inhibition of serum/CSF NFL concentration with therapeutic effect on CELs or MS relapses (4). These important observations raised a possibilityof using serum NFL (sNFL) measurements for managing MS in individual patients.

The clinical value of a laboratory test is assessed by sensitivity (i.e., the ability of the test to correctly identify people with the measured process) and specificity (i.e., the ability of the test to correctly identify people without the measured process). A large multicenter study with 286 paired serum/CSF NFL measurements in patients with MS demonstrated that CSF NFL (cNFL) has higher accuracy for predicting CELs or MS relapse (i.e., 75% specificity and 67% sensitivity with area under receiver operator characteristic [AUROC] curve 0.77) compared with sNFL (i.e., 80% specificity and 45% sensitivity with AUROC 0.66) (2, 3).

Because published cohorts showed only modest correlations between cNFL and sNFL (i.e., explaining between 40% and 60% of variance) (5–7), we asked whether we could improve accuracy of sNFL in predicting MS activity (i.e., CEL or relapse) and MS severity (i.e., rate of MS progression) by identifying physiological confounding factors that affect sNFL concentrations. Because cNFL more accurately identified patients with MS activity than sNFL, we hypothesized that by adjusting sNFL measurements for physiological covariates that may affect release of NFL from CNS axons, distribution volume of sNFL, and its metabolism using multiple linear regression models, we would derive a reproducible equation that would better approximate sNFL to cNFL values measured in parallel and therefore predict MS activity with enhanced accuracy. Although we could not identify publications that assessed accuracy of NFL in predicting MS severity, we expected that cNFL would have superior accuracy than sNFL and that adjusting for the same confounders would strengthen the accuracy of sNFL in predicting MS severity, too.

## Results

### Adjusting sNFL levels for confounding factors using a multiple linear regression model improves the correlation between measured and predicted cNFL.

We measured NFL levels in 1138 matching CSF and serum samples collected from 571 participants in 7 diagnostic groups: healthy donors (HD), relapsing-remitting MS (RRMS), primary progressive MS (PPMS), secondary progressive MS (SPMS), clinically isolated syndrome (CIS), noninflammatory neurological diseases (NIND), and other inflammatory neurological diseases (OIND). The NIND and OIND cohorts had evidence of CNS injury. The cohort was split into training (2/3) and validation (1/3) data sets prior to running any analysis, with a balanced distribution of each diagnosis in each data set. Because our first goal was to develop a generally valid mathematical adjustment that would better approximate sNFL levels to cNFL, we deliberately included patients with varied diagnoses.

**Figure 1:**
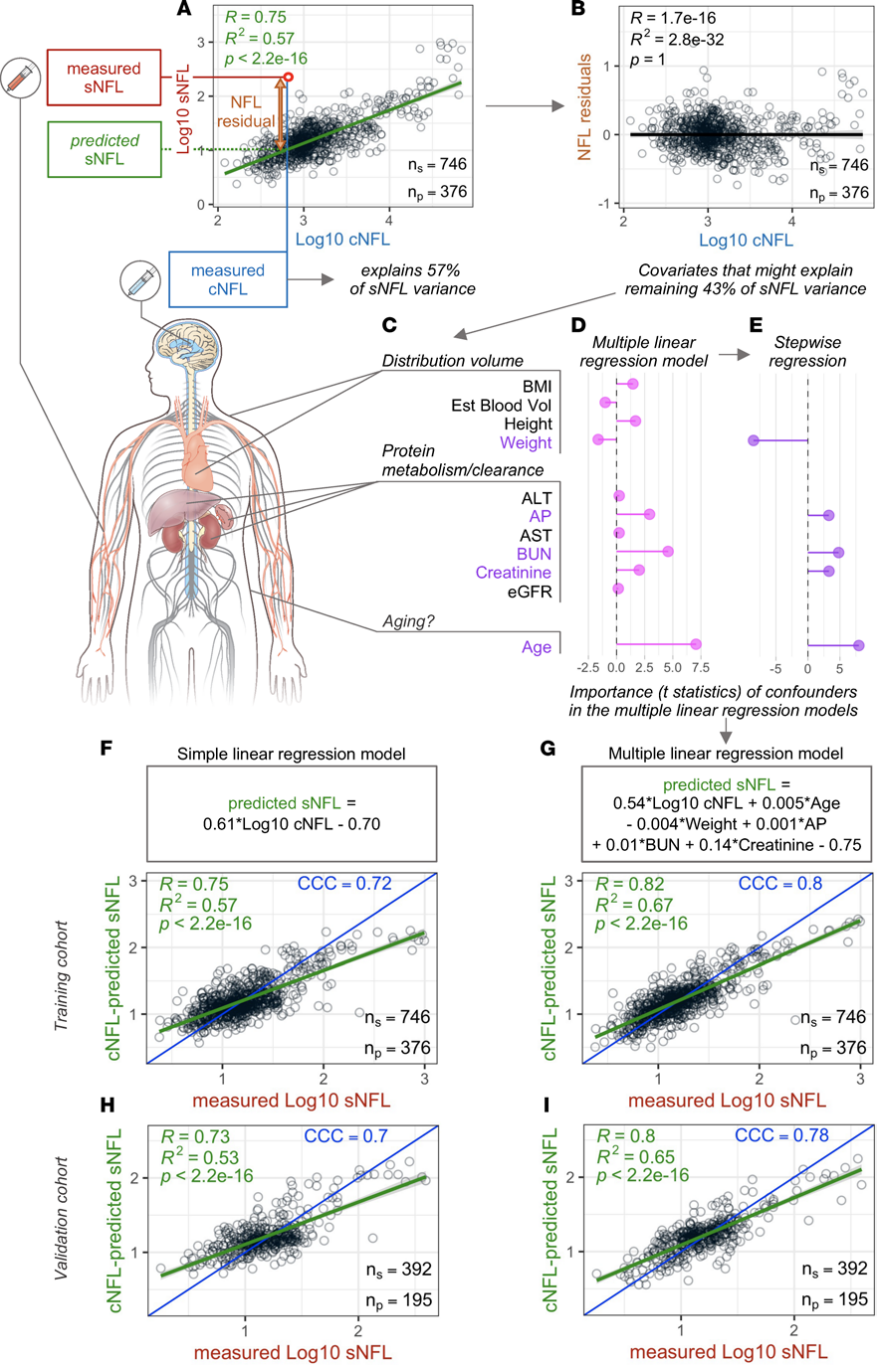
Variance between sNFL and cNFL concentrations. (**A**) Linear regression model between log_10_-transformed concentration (pg/mL) of sNFL and cNFL in the training cohort of samples where cNFL levels explain 57% of variance of sNFL levels. (**B**) Remaining 43% of variance shown as NFL residuals generated as differences between measured sNFL concentration and predicted sNFL concentration calculated from measured cNFL using linear regression model. (**C**) Eleven potential confounders related to distribution volume (BMI = body mass index, Est Blood Vol = estimated blood volume, height, and weight), protein metabolism/clearance (ALT = alanine transaminase, AP = alkaline phosphatase, AST = aspartate transaminase, BUN = blood urea nitrogen, creatinine, and eGFR = estimated glomerular filtration rate), and age were used as explanatory variables in a multiple linear regression model resulting in varied importance represented as a *t* statistic of each variable in the model (**D**). Stepwise regression resulted in retention of 5 confounders in the model (**E**) that showed increased correlation between measured and predicted sNFL levels both in the training (**G**) and in the validation (**I**) cohort in comparison with correlations between measured and predicted values using a simple linear regression model in the same training (**F**) and validation cohort (**H**). Confounders in color are the ones selected in the multiple linear regression model that underwent stepwise regression. Green line represents linear regression model with gray shading corresponding to 95% confidence interval. n_s_, number of samples measured; n_p_, number of patients represented by the samples; CCC, concordance correlation coefficient.

The correlation between sNFL and cNFL in the training cohort showed that cNFL explained 57% of the variance of sNFL (Figure 1A), which falls into the higher-end estimate from the published studies (5–7). To search for confounding factors responsible for the remaining 43% of variance between cNFL and sNFL (Figure 1B), we assumed that NFL is released from CNS axons into CSF, which is then drained (partially via lymph) to blood. Because of the prolonged dynamic of NFL release after acute injury, a steady state between cNFL and sNFL will ensue. We considered following confounding factors that may influence this steady state by modifying: a) release of NFL from the CNS (e.g., age); b) distribution volume of sNFL (i.e., reflected by body mass index [BMI], height, weight, and estimated blood volume) and c) sNFL metabolism/clearance from body (i.e., reflected by liver function tests: alanine transaminase [ALT], aspartate aminotransferase [AST]; by phagocytosis via the reticulo-endothelial system reflected by alkaline phosphatase [AP]; by protein metabolism reflected by blood urea nitrogen [BUN]; and by kidney clearance reflected by serum creatinine and estimated glomerular filtration rate [eGFR]; Figure 1C). Of these, the stepwise multiple linear regression (MLR) retained only weight, AP, BUN, creatinine, and age (Figure 1, D and E).

Although our goal is to adjust sNFL measurements to better approximate cNFL, the mathematical strategy to derive such an adjustment is counterintuitive: because we assume that the source of sNFL is CNS (all patients other than HD have CNS disease) and the route of NFL release is via CSF to blood, the sNFL concentration is cNFL modified by confounders. Without confounding factors, the sNFL would depend on only cNFL, which was approximated by a linear equation derived from the training cohort in Figure 1F, where it explained 57% of variance. When applied to the independent validation cohort (Figure 1H), this linear model explained 53% of the variance with a very low *P* value. In an MLR model, the sNFL concentration depended on cNFL, but its concentration was further modified by 5 confounders, reflected by the equation in Figure 1G derived from the training cohort, where it enhanced the proportion of variance explained from 57% to 67% (Figure 1F versus Figure 1G; 10% absolute and 15% relative gain in accuracy). This gain in accuracy was reproducible, with improvement from 53% to 65% of variance explained in an independent validation cohort (Figure 1H versus Figure 1I; 12% absolute and 18.5% relative gain in accuracy).

To ensure that longitudinal samples (i.e., multiple samples per patient) did not affect the model’s performance, we showed analogous validation in the cohort that contained only the first collected sample per patient or the median value of all samples per patient (Supplemental Figure 1; supplemental material available online with this article; https://doi.org/10.1172/jci.insight.161415DS1).

Reshuffling the equation from the final MLR model, we now have the correct adjustment of measured sNFL values that better predicts cNFL: sNFL-predicted cNFL = (log_10_ sNFL – [0.005 × age] + [0.004 × weight] – [0.001 × AP] – [0.01 × BUN] – [0.14 × creatinine] + 0.75)/0.54.

### Adjusted sNFL correlates better with MRI CELs than measured sNFL.

The most important question is whether the proposed mathematical adjustment enhances the clinical value of sNFL, including its ability to predict MS activity.

Thus, for all subsequent studies, we compared the effects of measured cNFL, measured sNFL, and sNFL-predicted cNFL (i.e., sNFL adjusted for validated confounders) on clinical and imaging outcomes in the MS cohort only.

**Figure 2:**
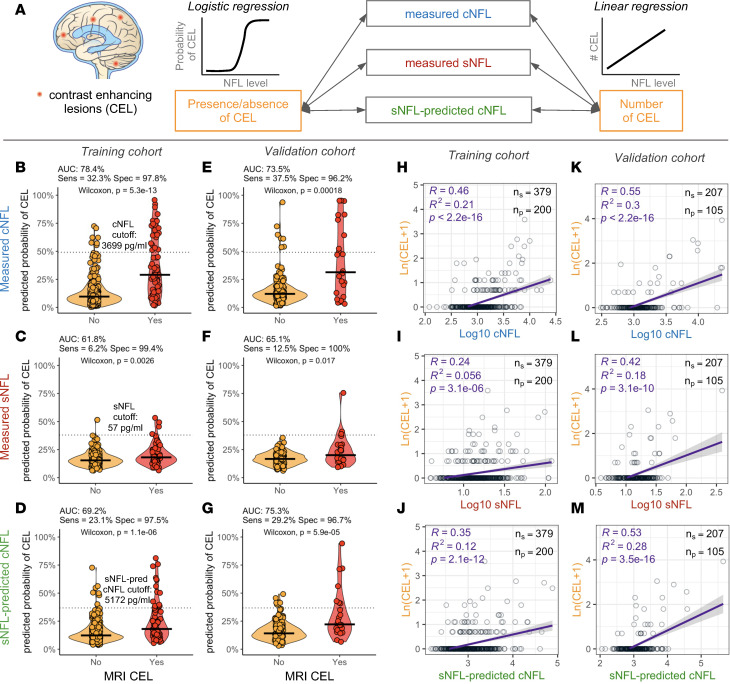
Adjustment for 5 confounders improves correlation of sNFL with number of MRI CELs and eliminates noise. (**A**) CELs have been used as a surrogate outcome of blood-brain barrier opening and active inflammation in the brains of patients with MS. Logistic regression that predicts probability of CEL presence/absence and linear regression between NFL and total number of CELs have been tested. A binomial regression classifier was generated to predict dichotomous outcome of present/absent CEL. The area under the curve (AUC), sensitivity, and specificity have been calculated for classifiers using measured cNFL (**B** and **E**), measured sNFL (**C** and **F**), and sNFL-predicted cNFL (**D** and **G**) to predict probability of presence of CELs. Dotted line represents the best probability cutoff value determined in the training cohort with corresponding NFL concentration displayed above the line. Horizontal lines represent medians. Two-sided Wilcoxon 2-sample test evaluated the significance of differences between 2 groups of patients. The linear model between number of CELs (*y* axes, transformed as natural logarithm of [CEL+1]) and NFL (*x* axes) shows higher predictive power of cNFL in both training (**H**) and validation (**K**) cohorts, compared with sNFL in training (**I**) and validation (**L**) cohorts. Adjustment of sNFL for 5 confounders (age, weight, AP, BUN, and creatinine) improved the correlation with number of CELs in both training (**J**) and validation (**M**) cohorts compared with measured sNFL. Purple line represents linear regression model with gray shading corresponding to 95% confidence interval.

There are 2 approaches for assessing MS activity. One is to dichotomize patients into those who do or do not have CELs or MS exacerbation at the time of NFL measurement and use the training cohort data to select the optimal NFL value based on area under the curve (AUC; Figure 2, A–G). This approach was used previously (2, 3) and is expanded here by assessing AUCs after applying NFL dichotomization cutoffs from the training cohort (measured cNFL = 3699 pg/mL, measured sNFL = 57 pg/mL, and sNFL-predicted cNFL = 5172 pg/mL; Figure 2, B–D) to the validation cohort. We reproduced the published observation that cNFL is a stronger predictor of MS activity as compared with sNFL in the training cohort: i.e., cNFL achieved AUC 78.4% versus 61.8% for sNFL (Figure 2, B and C). This hierarchy was validated in the independent cohort, where the AUC of cNFL was 73.5% and sNFL 65.1% (Figure 2, E and F). The mathematical adjustment of sNFL for confounding factors increased AUCs as compared with measured sNFL in both training (Figure 2C versus Figure 2D; from AUC 61.8% to 69.2%) and validation (Figure 2F versus Figure 2G; from AUC 65.1% to 75.3%) cohorts.

However, simply dichotomizing patients is suboptimal. The true clinical value of laboratory tests resides in their quantitative aspect. For example, dichotomizing liver function tests into normal and abnormal would not inform clinical care of the patients in the abnormal category. Therefore, we also assessed accuracy of NFL in estimating the level of disease activity, by generating models that predict the number of CELs in the independent validation cohort.

We observed that cNFL correlated stronger with number of CELs than sNFL. In linear regression models, cNFL explained 21% of variance of CELs (*P* < 2.2 × 10^–16^; Figure 2H), while sNFL explained 5.6% of variance (*P* = 3.1 × 10^–6^; Figure 2I) in the training cohort. Similar correlations were observed in an independent validation cohort (Figure 2, K and L). Adjusting sNFL for confounders (Figure 2, J and M) improved the correlation with the number of CELs in comparison with measured sNFL by increasing the variance explained from 5.6% to 12% in the training cohort (Figure 2I versus Figure 2J) and from 18% to 28% in an independent validation cohort (Figure 2L versus Figure 2M). This translates into relative improvement of 36% in the validation cohort.

Supplemental Figure 2 contains additional sensitivity analyses assessing exponential models and Poisson regressions that show analogous results. We also tested whether the improvement was statistically significant and observed significantly lower confounder-adjusted sNFL residuals of CELs from the Poisson regression models compared with unadjusted sNFL residuals (*P* = 0.003, paired Wilcoxon rank sum test).

This validates the hypothesis that adjusting measured sNFL levels for identified covariates meaningfully improves their ability to predict MS activity in an independent validation cohort.

### All NFL measurements are poor predictors of MS severity, but sNFL shows at least weak but significant correlations with MS severity outcomes.

As increased levels of NFL reflect MS-related acute CNS injury, with cNFL demonstrating higher accuracy than sNFL, we hypothesized that elevated NFL levels would be associated with faster accumulation of clinical disability and expected that cNFL would again demonstrate higher accuracy.

**Figure 3:**
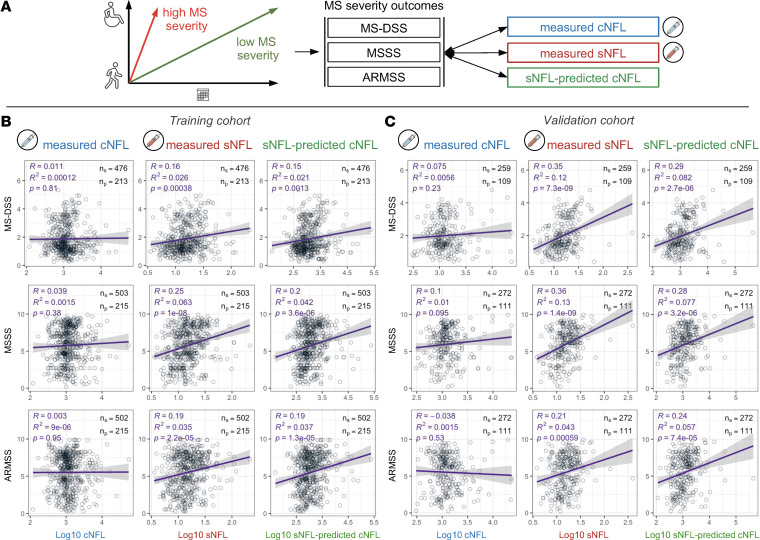
sNFL correlates better with MS disease severity outcomes than cNFL. (**A**) Disease severity in MS is a measure of how fast patients accumulate disability. Slow accumulation of disability over time results in low MS severity (green); fast accumulation of disability results in high MS severity (red). Because it is difficult to measure rates of disability progression prospectively and longitudinally, MS severity outcomes are collected cross-sectionally, measuring past rates of disability progression by normalizing disability to the patient’s age (Age-Related Multiple Sclerosis Severity Score [ARMSS] and Multiple Sclerosis Disease Severity Scale [MS-DSS]) or disease duration (MSSS). Correlation analysis of 3 MS severity outcomes, MS-DSS, MSSS, and ARMSS, with 3 NFL values, measured cNFL, measured sNFL, and sNFL-predicted cNFL, in 2 independent cohorts: training cohort (**B**) and validation cohort (**C**). Purple line represents linear regression model with gray shading corresponding to 95% confidence interval. Difference in number of patients/samples used for these analyses is because of exclusion of samples due to missing respective MS severity data.

MS-related clinical disability is traditionally measured by the ordinal Expanded Disability Status Scale (EDSS) (8). However, natural history studies show that patients with MS progress, on average, by approximately 1 EDSS point per decade. This has been validated in MS clinical trials, where approximately 10% of placebo-treated patients experience sustained disability progression on the EDSS for each 1 year of trial duration. Thus, the EDSS cannot correctly quantify patient-specific MS progression in observational studies with follow-up shorter than 10 years; most patients in our cohort had follow-up shorter than 10 years. Consequently, we assessed correlations between 3 NFL values and cross-sectional MS severity outcomes: Multiple Sclerosis Disease Severity Scale (MS-DSS) (9), Multiple Sclerosis Severity Scale (MSSS) (10), and Age-Related Multiple Sclerosis Severity (ARMSS) (11) (Figure 3). Note that these outcomes reflect past rates of disability accumulation by normalizing cross-sectional disability to patients’ age (ARMSS and MS-DSS) or disease duration (MSSS).

Contrary to our predictions, sNFL consistently outperformed cNFL (Figure 3). None of the severity outcomes showed a statistically significant correlation with cNFL, but all 3 showed a weak but statistically significant correlation with sNFL (Figure 3B). Specifically, unadjusted sNFL measurements explained 2.6% (MS-DSS), 6.3% (MSSS), and 3.5% (ARMSS) of MS severity variance in the training cohort. These unexpected observations were validated in an independent validation cohort, where sNFL explained 12% of MS-DSS, 13% of MSSS, and 4.3% of ARMSS (Figure 3C). As would be expected from the adjustment that better approximates sNFL to cNFL levels, adjusting sNFL for covariates generally weakened correlations with MS severity outcomes, although all remained statistically significant (Figure 3, B and C).

### Why sNFL correlates stronger with MS severity outcomes compared with cNFL.

**Figure 4:**
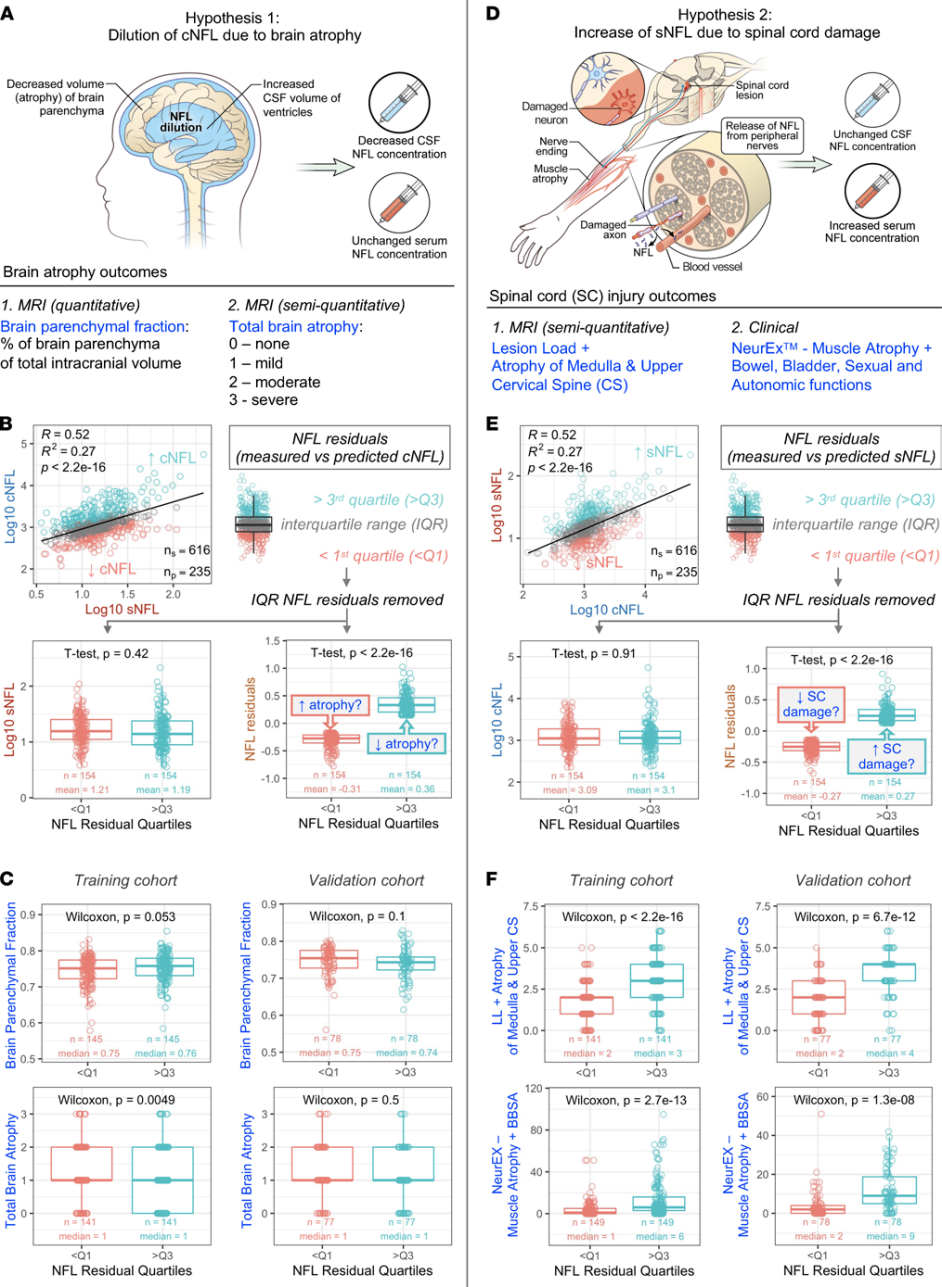
Two hypotheses explaining superiority of sNFL in predicting MS severity. (**A**) Hypothesis 1: Dilution of cNFL due to brain atrophy while sNFL concentration remains unaffected. Brain atrophy was evaluated by brain parenchymal fraction (BPFr) and by semiquantitative measure of brain atrophy (none, mild, moderate, and severe). (**B**) NFL residuals that fall within IQR (gray) were removed, resulting in a subset of samples with proportionally higher (above the third quartile [teal]) and lower cNFL (below the first quartile [salmon]), with comparable sNFL levels. (**C**) Paired Wilcoxon rank sum test showed marginally significant difference in BPFr (top left) and total brain atrophy (bottom left) between samples with different cNFL levels in the training cohort. These observations were not confirmed in the validation cohort (top and bottom right). (**D**) Hypothesis 2: Increase of sNFL due to spinal cord (SC) damage. NFL from damaged peripheral nerves and SC roots is released directly into blood, increasing sNFL concentration while cNFL remains unchanged. SC damage was evaluated using a semiquantitative MRI outcome (a sum of lesion load and atrophy at the level of medulla and cervical spine) and by clinical outcome capturing damage of lower motor neurons (sum of muscle atrophy scores) and damage to peripheral/autonomous nervous system (score for bowel, bladder, sexual, and autonomic dysfunctions) generated from neurological exams digitalized using the NeurEx app. (**E**) NFL residuals that fall within IQR (gray) were removed, resulting in a subset of samples with proportionally higher sNFL (above the third quartile [teal]) and lower sNFL (below the first quartile [salmon]), with comparable cNFL levels. (**F**) Paired Wilcoxon rank sum test showed a statistically significant difference in MRI (top left) and clinical (bottom left) outcomes between samples with different sNFL levels in the training cohort; the observed differences were confirmed in the validation cohort (top and bottom right). The box plots depict the minimum and maximum values (whiskers), the upper and lower quartiles, and the median. The length of the box represents the IQR.

We generated and tested 2 mutually nonexclusive hypotheses that may explain why sNFL correlates stronger with MS severity compared with cNFL: a) brain atrophy–associated MS progression leads to dilution of cNFL due to compensatory increase in CSF volume (Figure 4A) and b) spinal cord (SC) injury, associated with MS disability, such as injury to lower motor neurons or the autonomic nervous system, leads to release of NFL from axons of peripheral nerves into blood, bypassing the CSF (Figure 4D).

Both hypotheses were tested by focusing on patients with either comparable sNFL levels and highly different cNFL levels (testing hypothesis 1), or, conversely, comparable cNFL levels and highly different sNFL levels (testing hypothesis 2). We tested these using quartiles of appropriate sNFL-cNFL residuals (Figure 4, B and E) or by propensity score–matched samples (Supplemental Figure 3). Because both approaches provided analogous results, we present here only the simpler approach.

To test the hypothesis that increased CSF volume dilutes cNFL concentration, we asked whether patients with proportionally lower cNFL compared with sNFL concentrations have higher brain atrophy (and thus enlarged CSF volume) compared with patients with proportionally higher cNFL concentration. We tested 2 brain atrophy outcomes (Figure 4A): a) a fully quantitative BPFr measured retrospectively and b) prospectively acquired semiquantitative grading of the atrophy into 4 categories (none, mild, moderate, and severe) as part of a previously validated Combinatorial MRI scale (COMRIS) of CNS tissue destruction (12).

Consistent with the stated hypothesis, people with proportionally lower cNFL concentration had a marginally significant increase in brain atrophy measured by semiquantitative outcome (*P* = 0.0049) and BPFr (*P* = 0.053) in the training cohort. However, these weak differences were not validated in an independent validation cohort (Figure 4C).

To test the second hypothesis, we used prospectively acquired SC injury outcomes (Figure 4D). The first outcome was a semiquantitative assessment of lesion load and atrophy of the upper cervical SC graded from brain MRI images that extend to C5 level. This outcome has been previously validated as clinically meaningful (12, 13). Because it does not capture the damage to the thoracic or lumbosacral SC, we employed complementary information from NeurEx, which provides granular measurements of neurological disability (14). Two parts of NeurEx can be used for our purpose: grading of muscle atrophy, as a surrogate of injury to lower motor neurons and, by inference, to associated motor axons of peripheral nerves, as well as bowel, bladder, sexual, and autonomic (BBSA) dysfunctions, which in MS are likely caused by injury of the autonomic neurons that project axons to the autonomic ganglia.

Using these 2 outcomes, we observed a statistically significant increase in both imaging and clinical SC damage outcomes in the subgroup of patients with proportionally higher sNFL compared with cNFL, fitting the proposed model. These findings were robustly validated in an independent validation cohort (Figure 4F and Supplemental Figure 4).

We conclude MS-associated SC injury leads to release of NFL to blood, bypassing the CSF, likely because of Wallerian degeneration of peripheral axons. Thus, SC damage, which is a strong predictor of MS severity, is preferentially reflected by sNFL as compared with cNFL, leading to stronger correlation of sNFL with MS severity outcomes. This conclusion is supported by the fact that including SC damage outcomes (i.e., MRI SC atrophy, muscle atrophy, and BBSA) into the MLR model further strengthened the correlation between measured and predicted sNFL in an independent cohort (Supplemental Figure 5).

## Discussion

We started this work with the premise that cNFL is a clinically more relevant biomarker of CNS injury than sNFL and that we might enhance the clinical value of sNFL by adjusting for relevant confounders.

We validated this premise partially: we identified general confounders and validated an MLR model that adjusted sNFL to better approximate cNFL concentrations. The covariates selected by the MLR model are logical and affect sNFL concentration in a biologically predictable manner: high BUN, AP, and creatinine increase sNFL concentrations as they slow down NFL metabolism and clearance, while diluting NFL in larger distribution volume reflected by higher weight lowers sNFL concentration.

Some of these confounders (e.g., age, BMI and creatinine) were inferred in previous studies analyzing only sNFL in healthy volunteers (15, 16) or in patients with diabetes mellitus and renal dysfunction (16). However, lack of matched cNFL concentrations in these studies precludes eliminating the alternative explanation that these confounders, such as renal dysfunction, cause subclinical axonal damage, which increases sNFL. The current study rules out this alternative explanation and extends previous studies by identifying the most comprehensive set of confounders to our knowledge and showing that this comprehensive adjustment of sNFL values further improves their correlation with cNFL (Supplemental Figure 6). Most importantly, when we applied these adjustments to the MS cohort only, we observed that covariate-adjusted sNFL demonstrated a statistically significant increase in correlations with number of CELs and improved dichotomized prediction of MS activity when compared with measured sNFL. These improvements were validated in an independent validation cohort, where covariate-adjusted sNFL reached 36% relative enhancement of accuracy to predict presence of CEL. This is substantial improvement for a test aimed to be used in individual patients. These data validate and strengthen previous reports of using sNFL to identify patients with MS activity; in comparison with previously reported AUC = 0.66 for sNFL (measured in the training cohort only) (2, 3), we achieved AUC = 0.753 in an independent validation cohort for covariate-adjusted sNFL. Although this AUC approaches the accuracy of a clinically meaningful cross-sectional test, it should be noted that the test has high specificity (96.7%) but poor sensitivity (29.2%). Therefore, a positive test adds clinical value in identifying MS patients with disease activity, e.g., during monitoring of treatment efficacy. However, due to poor sensitivity, a clinician must supplement NFL measurements with CNS imaging, perhaps performed less frequently, to verify that prescribed treatment truly abrogates formation of new MS lesions.

The potentially novel and intriguing findings from our study are that sNFL outperformed cNFL in correlating with cross-sectional MS severity outcomes and that this is due to sNFL’s ability to capture NFL released from 2 sources: axons in the CNS but also from SC injury affecting lower motor neurons and the autonomic nervous system. The strength of our study resides in prospective acquisition of complementary clinical and imaging data that reliably capture SC injury and associated damage to lower motor and autonomic neurons. These data were locked into a database before any NFL measurements were collected, and NFL was measured in a blinded fashion by investigators who had no access to clinical or imaging data. The congruency of observations between training and validation cohorts and very low *P* values provide high confidence that our conclusions are valid. The fact that adding imaging and clinical outcomes of SC damage to the MLR model further strengthened the correlation between cNFL and sNFL shows that release of NFL from these SC sources is captured only by sNFL, not by cNFL. Although we logically infer that the source of NFL associated with imaging and clinical outcomes of SC injury is Wallerian degeneration of peripheral axons, we have not measured PNS injury in this study. Nevertheless, our conclusion, potentially novel for the MS field, is supported by the observation that patients with PNS disorders, such as Guillain-Barré syndrome, have proportionally higher sNFL to cNFL levels compared with patients with CNS disorders, such as Alzheimer’s disease, or compared with patients with combined involvement of CNS and peripheral axons, such as amyotrophic lateral sclerosis (17).

Despite its ability to (at least partially) reflect both brain and SC injury, the power of sNFL measurement to predict MS severity is, unfortunately, rather weak (i.e., explaining between 2.6% and 13% of variance of different MS severity outcomes). This observation suggests that sNFL will have limited value in guiding therapeutic decisions in patients who no longer form CELs. However, our data set is suboptimal for determining prognostic value of sNFL in MS: this is an observational, natural history cohort with duration of follow-up less than 10 years in most patients, which precluded us from evaluating the ability of sNFL to predict (future) rates of EDSS progression. Recently the ability of sNFL to predict disability progression was evaluated in 2 placebo-controlled phase III clinical trials of progressive MS: the EXPAND trial that evaluated efficacy of siponimod in SPMS and INFORMS trial that evaluated efficacy of fingolimod in PPMS (4). There, sNFL (the authors measured plasma NFL, which is comparable to sNFL) dichotomized to “low” (<30 pg/mL) and “high” (≥30 pg/mL) has significant predictive value to identify patients at risk of MS progression. Unfortunately, this study did not report AUC, sensitivity/specificity, and predictive values of dichotomized sNFL, which are necessary to assess clinical utility of a test. Patients with CELs constituted 21.6% of EXPAND and 10.1% of INFORMS participants, and in both trials, presence of CELs was the strongest predictor of elevated sNFL. Nevertheless, the subgroup analyses demonstrated that even patients without CELs had a statistically significant increase in the risk of disability progression if they had high sNFL. But this study analyzed thousands of sNFL samples and achieved marginal *P* values for patients without CELs (e.g., *P* = 0.0274 for *n* = 1147 predicting 3 months confirmed disability progression in EXPAND trial). Because both effect size and number of participants contribute to *P* value, the marginal *P* value can be obtained from such large cohorts only if the effect size is very low. Thus, these data, generated from gold standard clinical trials, support our conclusion that while sNFL correlates with MS severity at a group level, its accuracy is likely too low to be clinically meaningful at an individual patient level. We discourage clinicians from interpreting our data, or (concurring) group data from other publications, as proof that sNFL provides actionable insight on a patient when caring for people with MS who no longer form CELs.

The final question is why sNFL does not predict MS progression with much higher accuracy when it is likely that neuronal loss causes development of brain atrophy and MS disability. Loss of neurons must lead to loss of axons, so why do many people with progressive MS have sNFL levels indistinguishable from HD? We speculate that the answer lies in the spatiotemporal dynamics of axonal damage. Pathology studies show concentrated axonal transections inside acute MS lesions but not outside of them. Such temporary and spatially concentrated axonal transections must release large amounts of NFL that likely overwhelms local phagocytes, leading to spillover of NFL into circulation. But if neuronal loss is distributed over a large CNS volume, microglia may have time to phagocytose dissolving axons and the NFL spillover is minimized. The type of neuronal death is probably also important: inflammation causes axonal transections associated with NFL release, whereas orderly apoptosis retains neuronal molecules encapsulated by membranes before they are phagocytosed.

Obviously, these are only speculations and are difficult to investigate in living systems. Nevertheless, our study and thus far all reported NFL studies in MS demonstrate that nonphysiological increase in NFL preferentially reflects acute axonal injury associated with formation of focal MS lesions. NFL levels are rather insensitive for capturing slow neuro-axonal loss associated with MS progression in people who no longer form CELs. If NFL should play a role in clinical care of such patients with MS, we need publications that quantify the predictive value of NFL on an individual patient level.

## Methods

### Patients.

Matching CSF and serum samples (1138 each) were prospectively collected from 571 patients (Table 1) from 7 diagnostic categories: HD, RRMS, PPMS, SPMS, CIS, NIND, and OIND. Collected samples were split into training (2/3) and validation (1/3) cohorts, controlling for diagnoses and keeping longitudinal samples in the same cohort to ensure complete independence of the participants in 2 cohorts.

All laboratory, clinical, and MRI outcomes (see Supplemental Methods) were prospectively collected in a database, quality controlled during weekly clinical care meetings, and locked to prevent further modifications. These data were collected prior to blinded evaluation of CSF and serum NFL levels from cryopreserved samples.

### Laboratory, clinical, and MRI outcomes.

Height and weight measurements were taken, and laboratory tests were performed at CSF/blood collection at the NIH Department of Laboratory Medicine and recorded in the NIH electronic medical records. MS patients underwent a full neurological exam and brain MRI at sample collection. The neurological exam was documented electronically using the NeurEx app (14) that contains algorithms calculating traditional disability scales (e.g., EDSS, including Kurtzke Functional System Scores) that eliminate noise stemming from inaccuracy of translating neurological examination into disability scales by clinicians. The research brain MRI (with or without gadolinium contrast) was performed on 1.5 T (Signa; GE Healthcare) and 3 T (MAGNETOM Skyra; Siemens) scanners. MRI sequences included T1 magnetization–prepared rapid gradient echo or fast spoiled gradient echo and T2-weighted 3-dimensional fluid attenuation inversion recovery sequences that were reviewed and graded by a board-certified neurologist and recorded using the previously published COMRIS tool (12) on a research database. The brain MRI protocol used extends sagittal and axial cuts distally to the C5 level, allowing determination of semiquantitative MRI biomarkers of medulla/upper SC atrophy and lesion load. The quantitative MRI outcomes (e.g., brain parenchymal fraction) were generated using the cloud-based medical image-processing platform, QMENTA, using the LesionTOADS algorithm (18). MS severity outcomes per MS-DSS, MSSS, and ARMSS were calculated as described (9–11). Although MSSS and ARMSS are both based on EDSS related to disease duration and age, respectively, MS-DSS is a more complex, machine learning–based model with the strongest variable being combinatorial weight-adjusted disability score/age (19).

### Sample collection.

Samples were collected following the laboratory standard operating procedures. Briefly, CSF, collected by lumbar puncture, was kept on ice and processed within 15 minutes of collection by centrifugation at 335*g* for 10 minutes at 4°C; the supernatant was aliquoted and stored at –80°C. Blood was collected by venipuncture (SST tube), incubated at room temperature for 30 minutes, and spun at 2000*g* for 10 minutes at 4°C. The aliquoted serum was stored at –80°C. Personnel processing samples were blinded to patients’ diagnoses/clinical/MRI outcomes.

### cNFL ELISA.

As NFL concentration in CSF is higher (~10- to 100-fold higher than sNFL), it can be reliably measured with a comparatively less sensitive assay, such as ELISA. We measured cNFL concentrations using solid-phase sandwich ELISA (UmanDiagnostics, catalog number: 10-7002 RUO; lower limit of detection [LLoD]: 33 pg/mL; see Supplemental Methods).

All samples were diluted 1:2 with provided sample diluent and then analyzed blindly. Samples were analyzed on multiple plates; location of samples on each plate was randomized, and a control sample was analyzed on each plate. The coefficient of variance (CV) for the control sample across all plates was 6.6%, confirming the assay precision and reproducibility.

### sNFL SIMOA assay.

Serum NFL levels were analyzed using a SIMOA assay kit (Quanterix; product number: 103186; LLoD: 0.038 pg/mL) on a SIMOA HD-1 analyzer (see Supplemental Methods).

All samples were diluted 1:4 with provided sample diluent using onboard dilution functionality, then analyzed blindly. Samples were analyzed in 2 batches (batch 1: 12 plates and batch 2: 4 plates); each plate contained 2 quality control (QC) samples provided with the kit, 1 for low (C1) and 1 for high (C2) concentration. The CVs for measured concentrations of QC samples were within the acceptable range (batch 1: C1 = 9.8%, C2 = 9.8%; batch 2: C1 = 9.0%, C2 = 7.7%), confirming the assay precision.

Though we have used 2 different assays, ELISA and SIMOA, to assess CSF and serum, respectively, both assays use identical antibodies. ELISA is cheaper and has sufficient sensitivity for measuring cNFL, while the enhanced sensitivity of SIMOA assay is necessary to reliably measure sNFL. To ensure that no bias was introduced by using different assays for NFL measurements, we measured 68 CSF samples by both assays and confirmed that they yielded identical results (*R*^2^ = 0.97, *P* < 0.001; Supplemental Figure 7).

### Statistics.

All modeling/analyses/plots were performed in R Studio Version 1.1.463 (R version 4.0.2) (20). Simple and MLR models were generated using *lm* function (“stats” package; ref. 20). Correlations between variables were assessed using *stat_cor* function (“ggpubr” package; ref. 21), generating Pearson correlation coefficient (*r*), CV (*R*^2^), and *P* value. CCC was calculated using *epi.CCC* function of the “epiR” package. The final MLR model was selected using stepwise algorithm in *stepAIC* function (“MASS” package; ref. 22). Differences between groups were evaluated by *stat_compare_means* function (“ggpubr” package; ref. 21) using unpaired 2-sided *wilcox.test* or *t.test* method.

Prediction models of MRI CEL were developed using logistic regression (*glm* function of the “stat” package; ref. 20). Optimal cutoff for the predictive models was calculated using the *optimalCutoff* function of the “InformationValue” package (https://cran.r-project.org/web/packages/InformationValue). The AUROC was calculated using the *roc* function of the “pROC” package (23), and the specificity and sensitivity were calculated using specificity and sensitivity functions of the “caret” package (https://cran.r-project.org/web/packages/caret). The NFL cutoffs depicted in Figure 2, B–D, were calculated as mean of the highest NFL value below the optimal model cutoff and the lowest NFL value above the optimal model cutoff.

To test whether brain atrophy can explain superiority of sNFL over cNFL, we generated NFL residuals by subtracting the variance of cNFL explained by sNFL. Then we calculated quartiles of the NFL residual and removed samples falling within the IQR. Samples with NFL residuals below the first quartile represented patients with measured cNFL much lower than what would be predicted by the simple linear regression model. To test whether SC damage could explain superiority of sNFL in predicting MS severity, we generated NFL residuals, by subtracting variance of the sNFL explained by the measured cNFL. Then we eliminated samples with NFL residuals within the IQR, resulting in a group of samples with measured sNFL higher than what would be predicted by the model and samples with measured sNFL levels that were lower than what the model predicted. Differences between the samples from the first and the third quartile were evaluated using unpaired 2-tailed *wilcox.test* or *t.test* method.

Propensity score matching was performed using *matchit* function with “full” method (“MatchIt” package; ref. 24). Differences between propensity score–matched groups were evaluated by *stat_compare_means* function (“ggpubr” package; ref. 21) using paired 2-tailed *wilcox.test* or *t.test* method.

Poisson regression models were generated using *glm* function.

Although we provide raw *P* values that have not been adjusted for multiple comparisons, all *P* values in the independent validation cohort would remain significant after the most conservative Bonferroni adjustment. *P* < 0.05 was considered statistically significant.

The raw data and R code are provided as Supplemental Data 1.

### Study approval.

All samples were collected as part of a natural history protocol, “Comprehensive Multimodal Analysis of Neuroimmunological Diseases of the Central Nervous System” (ClinicalTrials.gov Identifier: NCT00794352), or as part of the “NIB Repository Protocol” (10-N-0210). The protocols were approved by the NIH Institutional Review Board. All participants signed a written informed consent document.

## Author contributions

BB designed and supervised the study. PK, RM, MK, JP, VR, MV, MS, and BB contributed to acquisition and analysis of the data. BB, PK, and RM drafted the text and prepared the figures. PK and RM share first authorship, listed in the authors list as per alphabetical order of last names.

## Supplementary Material

Supplemental data

ICMJE disclosure forms

Supplemental data set 1

Supplemental data set 2

Supplemental data set 3

## Figures and Tables

**Table 1:**
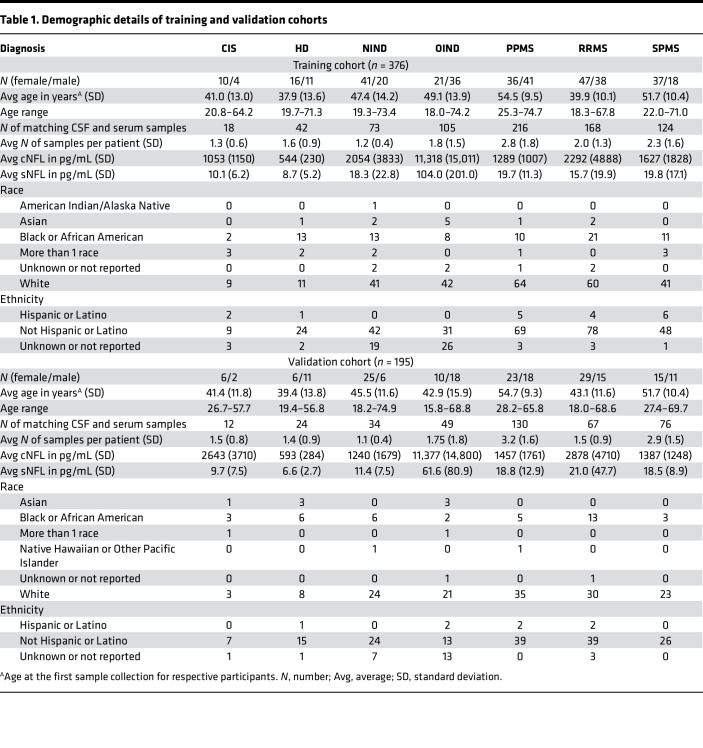
Demographic details of training and validation cohorts

## Version 1 06/23/2022

In-Press Preview

## Version 2 08/08/2022

Electronic publication
