# Supplementary material for: Enhancing the clinical value of serum neurofilament light chain measurement
Source: JCI Insight. 2022 Aug 8;7(15):e161415. doi: 10.1172/jci.insight.161415 (PMC9462467; doi:10.1172/jci.insight.161415)
Supplement: Supplemental data [file jciinsight-7-161415-s061.pdf]

Supplementary Methods

Laboratory, clinical, and MRI outcomes

Body measurements were taken, and laboratory tests were performed at the time of CSF/blood collection at the NIH Department of Laboratory Medicine and recorded in the NIH electronical medical records. MS patients underwent a full neurological exam and brain MRI at the time of sample collection. The neurological exam was documented electronically using NeurEx^{TM} App (1) that contains algorithms calculating traditional disability scales (e.g. Expanded Disability Status Scale [EDSS], including Kurtzke functional system scores) that eliminate noise stemming from inaccuracy of translating neurological examination into disability scales by clinicians. The research brain MRI (with or without gadolinium contrast) was performed on 1.5T and 3T scanners. MRI sequences included T1-magnetization-prepared rapid gradient-echo (MPRAGE) or fast spoiled gradient echo (FSPGR) and T2-weighted three-dimensional fluid attenuation inversion recovery (3D FLAIR) sequences that were reviewed and graded by a board-certified neurologist and recorded using previously published Combinatorial MRI Scale of CNS tissue destruction (COMRIS) tool (2) into research database. The brain MRI protocol used extends sagittal and axial cuts distally to C5 level, allowing determination of semi-quantitative (semi-qMRI) MRI biomarkers of medulla/upper spinal cord (SC) atrophy and lesion load. The quantitative MRI outcomes (e.g., brain parenchymal fraction) were generated using cloud-based medical image-processing platform, QMENTA, using LesionTOADS algorithm(3). MS severity outcomes – MS Disease Severity Scale (MS-DSS), MS Severity Score (MSSS), and Age-related MS Severity Score - were calculated as described (4-6). While MSSS and ARMSS are both based on EDSS related to disease duration and age, respectively, MS-DSS is a more complex,

machine learning-based model with the strongest variable being Combinatorial weight-adjusted disability score (CombiWISE (7))/Age.

NFL ELISA

All samples were diluted 1:2 with provided sample diluent and then analyzed blindly and in singlets. Samples were analyzed on multiple plates; location of samples on each plate was randomized and a control sample was analyzed in duplicate. The coefficient of variance (CV) for the control sample across the 12 plates was 6.6%, confirming the assay precision and reproducibility.

NFL SIMOA^{TM}

All samples were diluted 1:4 with provided sample diluent using on board dilution functionality, and then analyzed blindly in singlets. Samples were analyzed in two batches (batch 1: 12 plates and batch 2: 4 plates); each plate contained two quality control (QC) samples provided with kit, one for low (C1) and one for high (C2) concentration. The CVs for measured concentrations of QC samples were within acceptable range (batch 1, C1=9.8%, C2=9.8%; batch 2, C1=9.0%, C2=7.7%), confirming the assay precision.

Statistical Analysis

To test whether brain atrophy can explain superiority of sNFL over cNFL we generated NFL residuals by subtracting the variance of cNFL explained by sNFL. Then we calculated quartiles

of the NFL residual and removed samples falling within the interquartile range (IQR). Samples with NFL residuals below the first quartile represented patients with measured cNFL much lower than what would be predicted by the simple linear regression model. To test whether spinal cord damage could explain superiority of sNFL in predicting MS severity, we generated NFL residuals, by subtracting variance of the sNFL explained by the measured cNFL. Then we eliminated samples with NFL residuals within the IQR, resulting in a group of samples with measured sNFL higher than what would be predicted by the model and samples with measured sNFL levels that were lower than what the model predicted. Differences between the samples from the first and the third quartile were evaluated using unpaired *wilcox.test* or *t.test* method.

Propensity score matching was performed using *matchit* function with "full" method ("MatchIt" package(8)). Differences between propensity score-matched groups were evaluated by *stat_compare_means* function ("ggpubr" package (9)) using paired *wilcox.test* or *t.test* method.

Poisson regression models were generated using *glm* function ("stats" package {, 2020 #15}).

References

1. Kosa P, Barbour C, Wichman A, Sandford M, Greenwood M, and Bielekova B. NeurEx: digitalized neurological examination offers a novel high-resolution disability scale. *Ann Clin Transl Neurol.* 2018;5(10):1241-9.

2. Kosa P, Komori M, Waters R, Wu T, Cortese I, Ohayon J, et al. Novel composite MRI scale correlates highly with disability in multiple sclerosis patients. *Mult Scler Relat Disord.* 2015;4(6):526-35.

3. Shiee N, Bazin PL, Ozturk A, Reich DS, Calabresi PA, and Pham DL. A topology-preserving approach to the segmentation of brain images with multiple sclerosis lesions. *Neuroimage.* 2010;49(2):1524-35.

4. Manouchehrinia A, Westerlind H, Kingwell E, Zhu F, Carruthers R, Ramanujam R, et al. Age Related Multiple Sclerosis Severity Score: Disability ranked by age. *Mult Scler.* 2017;23(14):1938-46.

5. Petzold A, Eikelenboom MI, Keir G, Polman CH, Uitdehaag BM, Thompson EJ, et al. The new global multiple sclerosis severity score (MSSS) correlates with axonal but not glial biomarkers. *Mult Scler.* 2006;12(3):325-8.

6. Weideman AM, Barbour C, Tapia-Maltos MA, Tran T, Jackson K, Kosa P, et al. New Multiple Sclerosis Disease Severity Scale Predicts Future Accumulation of Disability. *Front Neurol.* 2017;8:598.

7. Kosa P, Ghazali D, Tanigawa M, Barbour C, Cortese I, Kelley W, et al. Development of a Sensitive Outcome for Economical Drug Screening for Progressive Multiple Sclerosis Treatment. *Front Neurol.* 2016;7:131.

8. Ho DE, Imai K, King G, and Stuart EA. MatchIt: Nonparametric Preprocessing for Parametric Causal Inference. . *Journal of Statistical Software.* 2011;42(8):1-28.

9. Kassambara A. ggpubr: 'ggplot2' Based Publication Ready Plots. R package version 0.4.0. https://CRAN.R-project.org/package=ggpubr.

Supplementary Figures

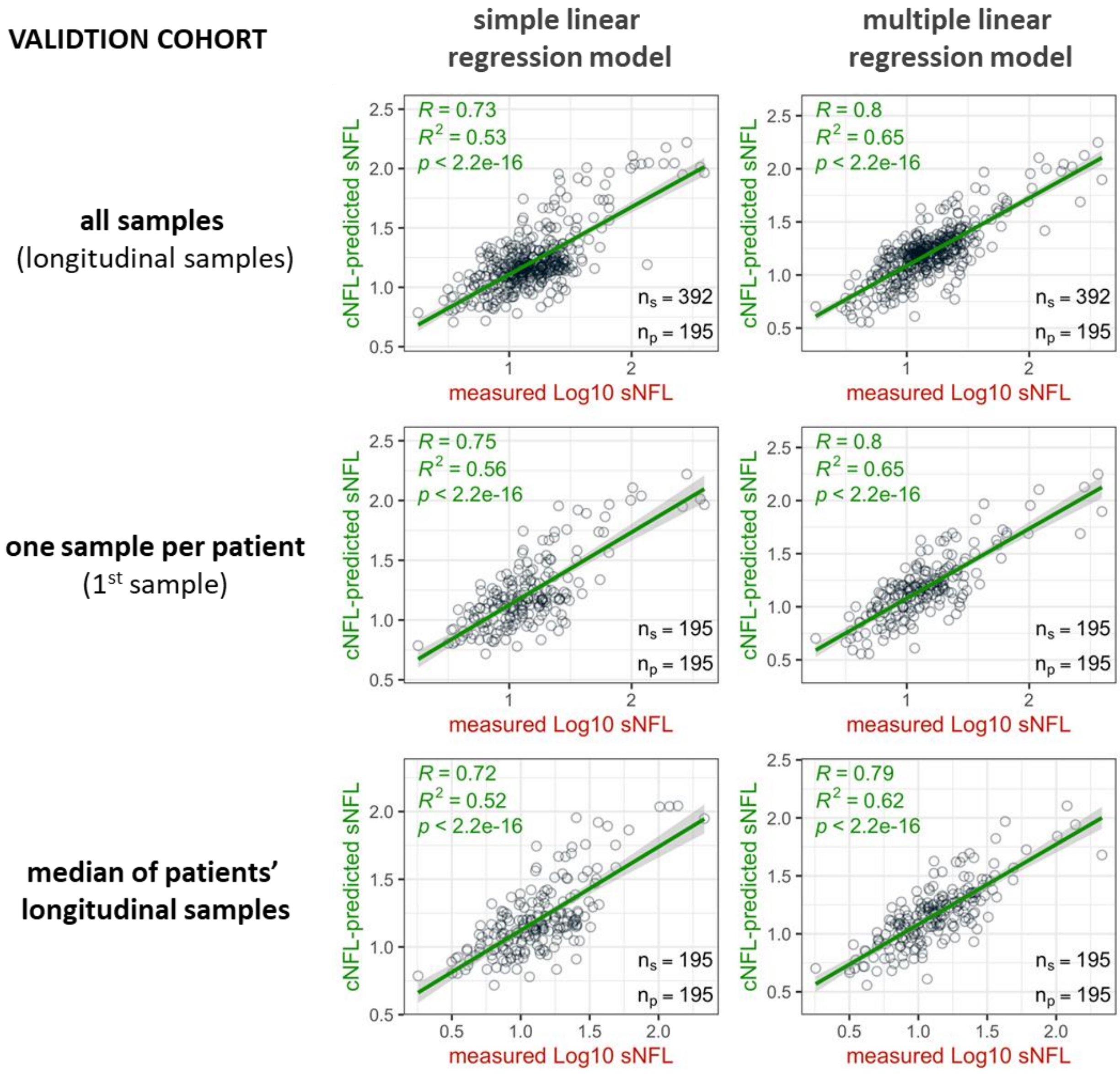

Supplementary Figure 1: Comparison of performance between simple linear regression model (left) and multiple linear regression model (right) in the validation cohort shows similar amount of variance explained between measured and predicted sNFL levels if all longitudinal samples were included (top), if only first sample per patient was included (middle), and if medians of NFL levels for longitudinal samples were considered (bottom). R – Pearson correlation coefficient, R^2 – coefficient of determination, ns - number of samples, np – number of patients.

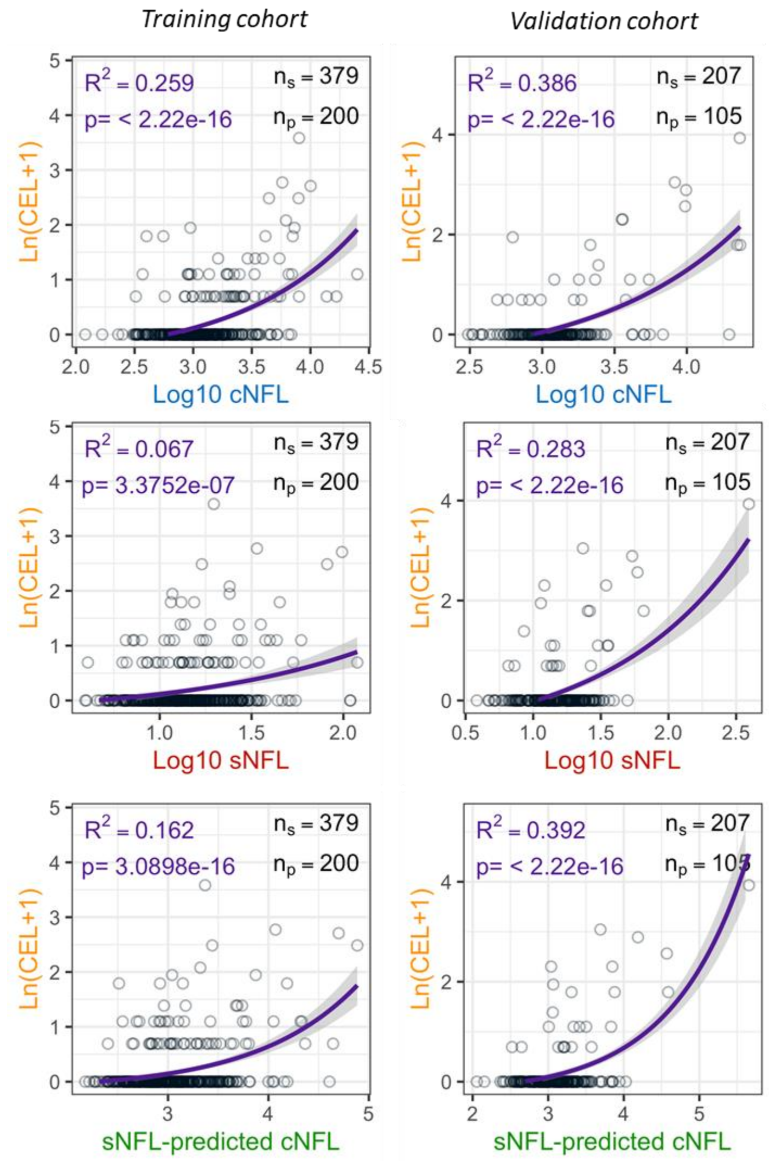

Supplementary Figure 2: Exponential fit (purple curve, gray shaded area represents 95% confidence interval) between measured cNFL, measured sNFL, and adjusted sNFL and the number of contrast enhancing lesions (CELs) expressed as natural logarithm on y axis shows the highest proportion of variance of CELs explained by cNFL, followed by adjusted sNFL, and the lowest by measured sNFL. R^2 – coefficient of determination, ns - number of samples, np – number of patients.

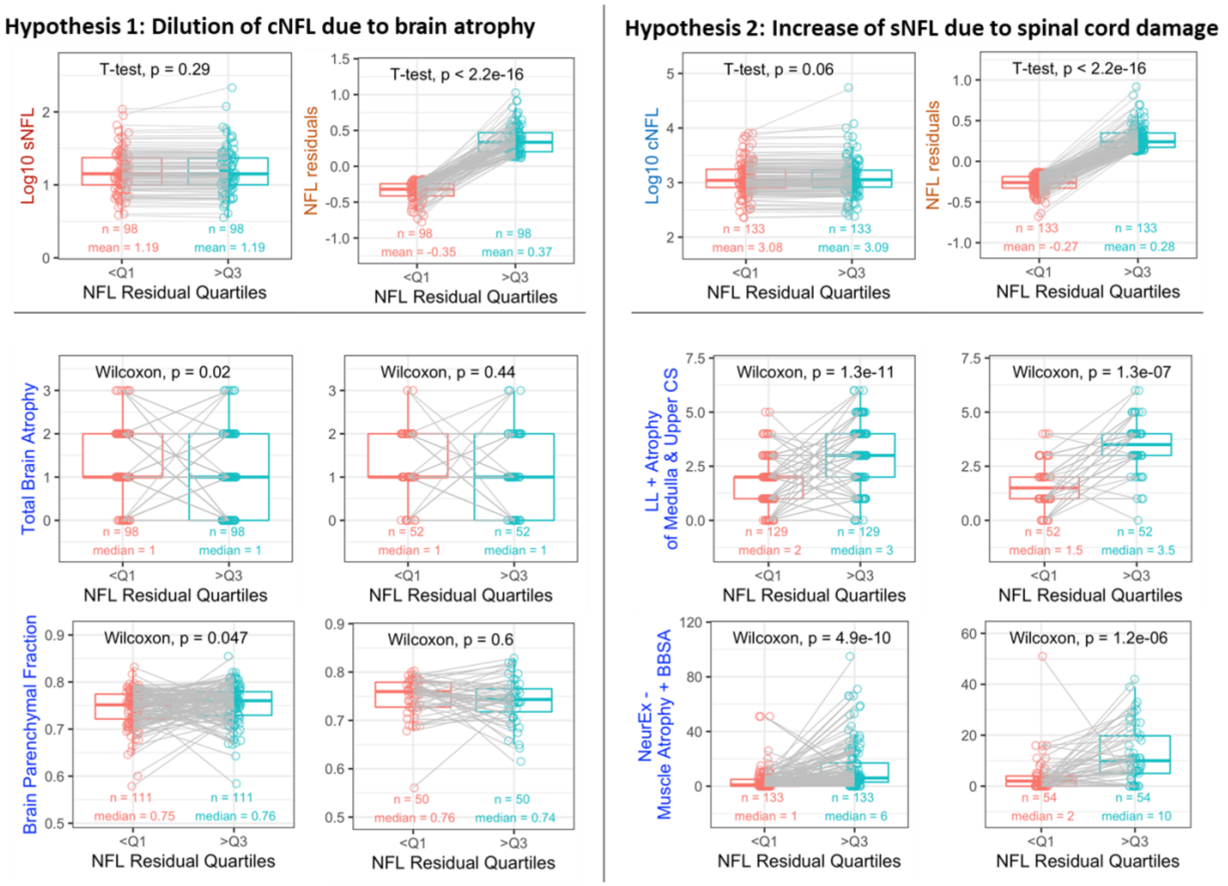

Supplementary Figure 3: Testing of two mutually non-exclusive hypotheses for sNFL's superiority compared to cNFL, in predicting MS severity in propensity score-matched datasets. NFL residuals from the first and the third quartile were matched for their sNFL levels (for Hypothesis 1) and for their cNFL levels (for Hypothesis 2). For hypothesis 1 (left), the matched pairs of samples were then evaluated using paired Wilcoxon signed rank test to ask whether there is a statistically significant difference in brain atrophy measured by semiquantitative total brain atrophy outcome and fully quantitative brain parenchymal fraction. Statistically significant increase in brain atrophy in samples with proportionally lower cNFL concentration in the training cohort failed to validate in an independent validation cohort. For hypothesis 2, the matched pairs of samples were evaluated using paired Wilcoxon signed rank test to ask whether there is a statistically significant difference in brain atrophy measured my semiquantitative MRI

outcome consisting of lesion load (LL) and atrophy of Medulla and upper cervical spine (CS) and by clinical outcome generated from neurological examination (NeurEx) assessing muscle atrophy and bowel, bladder, sexual and autonomic (BBSA) functions. Samples with proportionally higher sNFL levels compared to cNFL levels showed statistically significant increase in both outcomes measuring spinal cord damage in both training and validation cohorts.

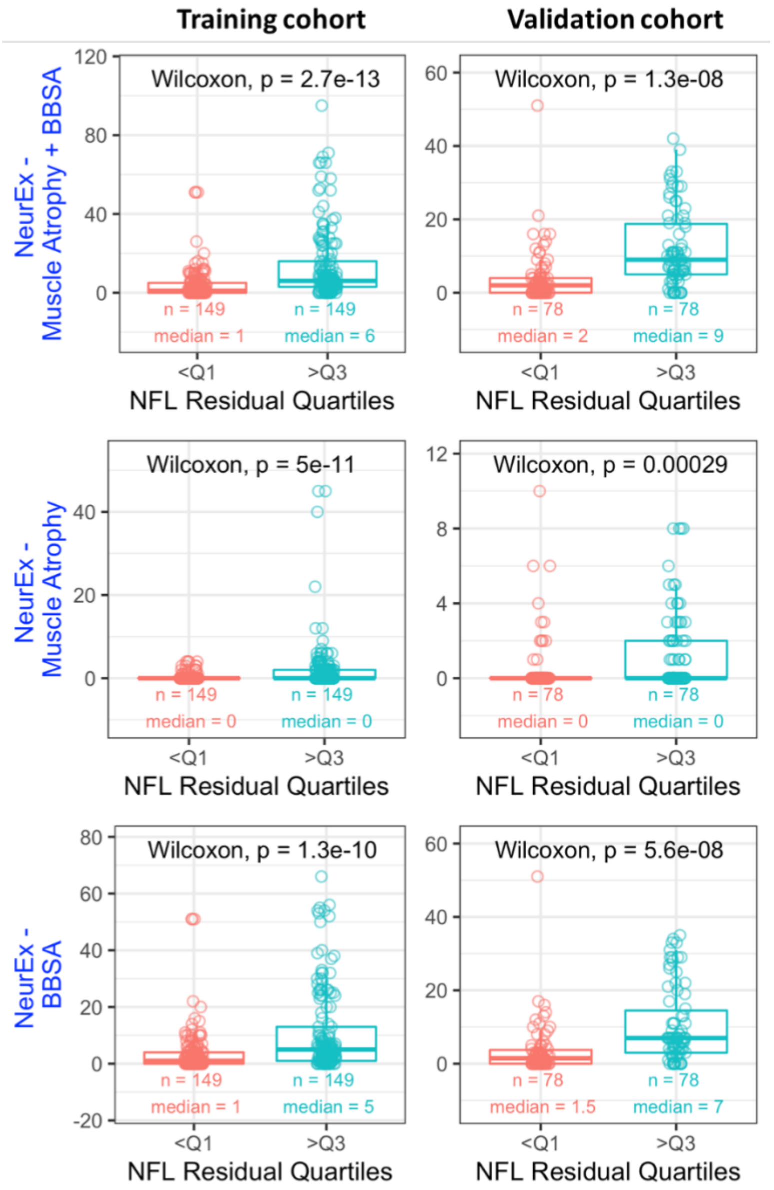

Supplementary Figure 4: Paired Wilcoxon Ranked Sum Test showed statistically significant difference in two parts of NeurEx^{TM} (muscle atrophy and bowel, bladder, sexual, and autonomic [BBSA] dysfunctions) combined (first row) or separated (muscle atrophy: second row and BBSA: third row) outcomes between samples with different sNFL levels in the training cohort; the observed differences were confirmed in the validation cohort.

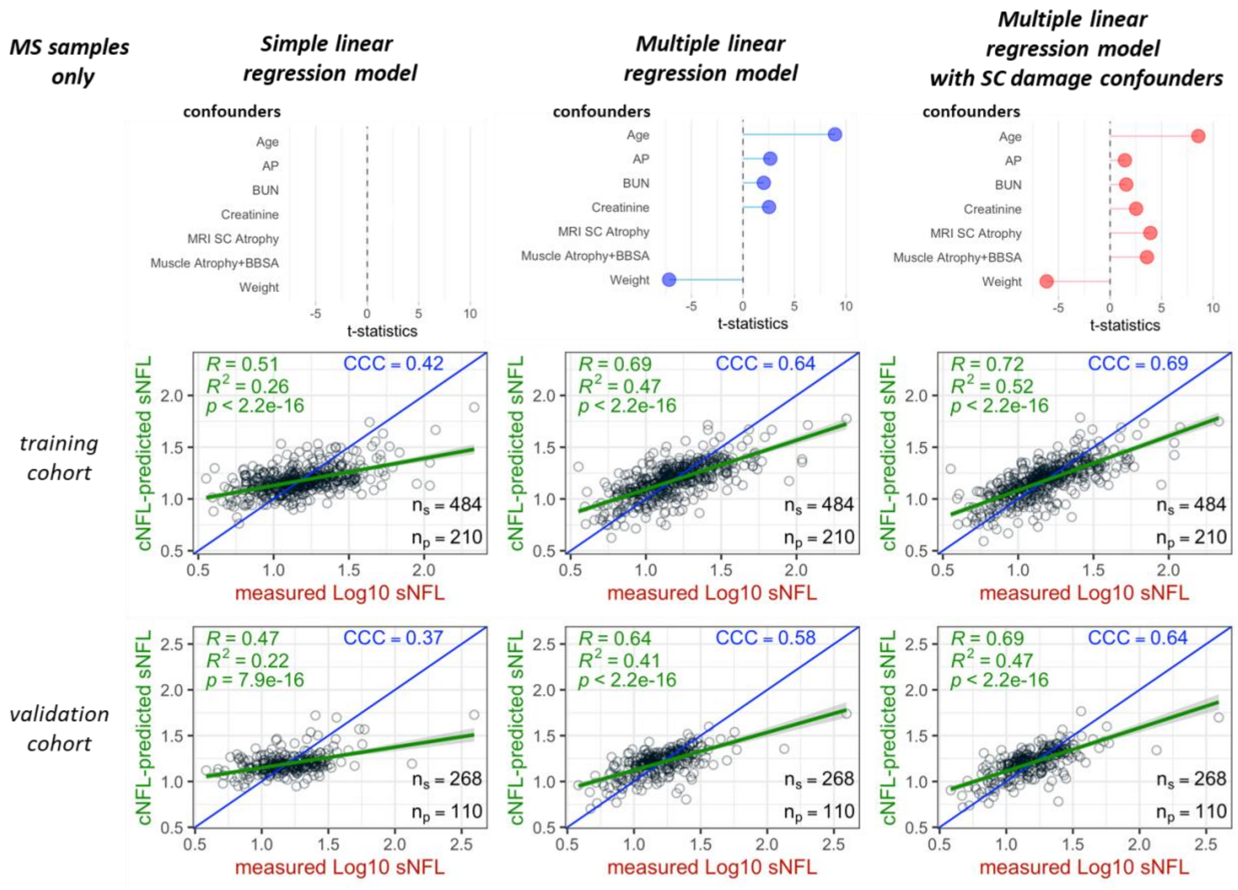

Supplementary Figure 5: Comparison of performance of simple linear regression model, multiple linear regression model including 5 validated confounders: age, alkaline phosphatase (AP), blood urea nitrogen (BUN), creatinine, and weight, and multiple linear regression model that includes two additional spinal cord (SC) damage outcomes: MRI SC atrophy and NeurEx-based muscle atrophy and bowel, bladder, sexual and autonomic (BBSA) dysfunction. In MS

samples only shows that addition of 5 confounders increased variance explained from 26% to 48% in the training cohort and from 22% to 41% in the validation cohort. Addition of SC damage outcomes further improved the model performance, increasing the variance explained to 53% and 47% in the training and validation cohorts, respectively. R – Pearson correlation coefficient, R^2 – coefficient of determination, CCC – concordance correlation coefficient, n_s - number of samples, n_p – number of patients.

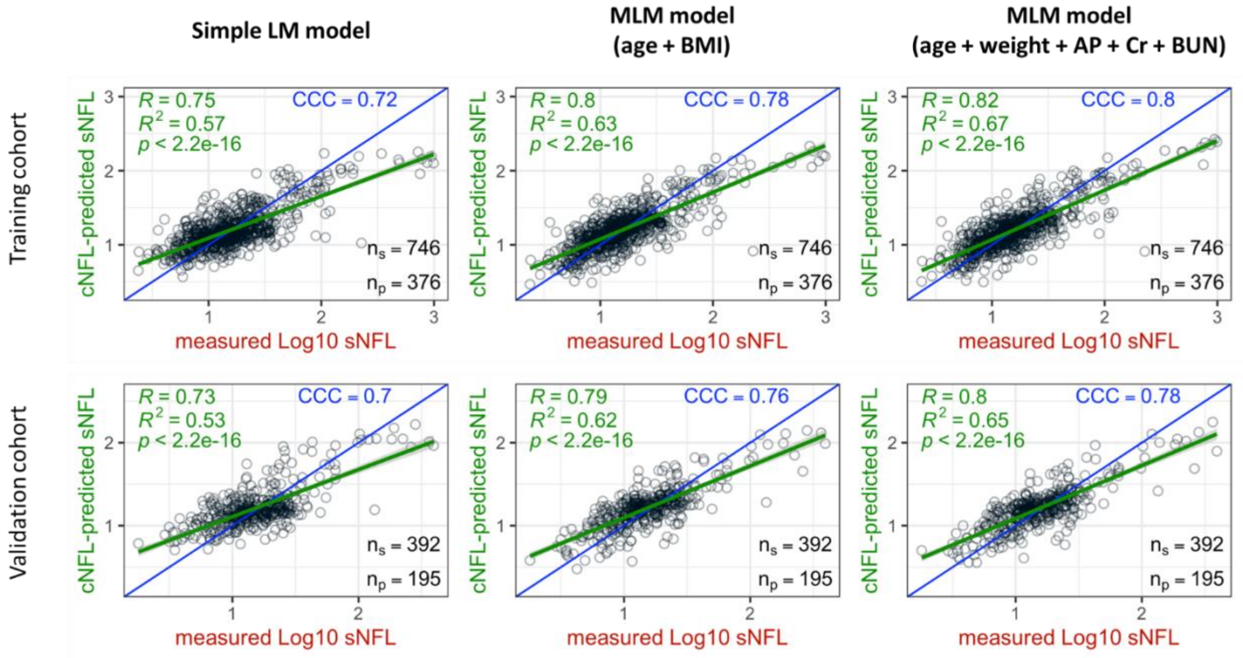

Supplementary Figure 6: Comparison of performance of simple linear regression model and multiple linear regression models including either just 2 confounders (age + BMI) or 5 confounders (age + weight + AP + Cr + BUN). R – Pearson correlation coefficient, R^2 – coefficient of determination, CCC – concordance correlation coefficient, ns – number of samples measured, np – number of patients represented by the samples. Green line represents linear regression model with gray shading corresponding to 95% confidence interval.

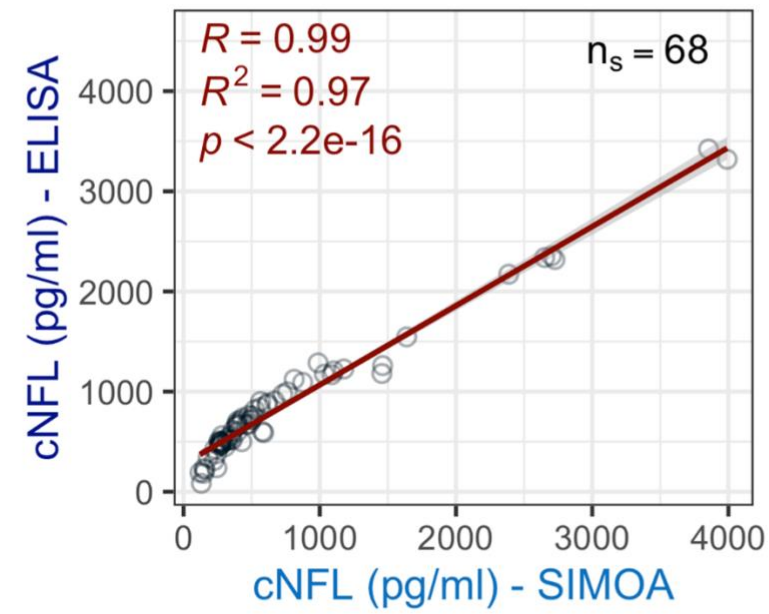

Supplementary Figure 7: Correlations between cNFL measurements using two different assays, ELISA and SIMOA. R – Pearson correlation coefficient, R^2 – coefficient of determination, n_s – number of samples measured. Brown line represents linear regression model with gray shading corresponding to 95% confidence interval.
